# Transcutaneous Auricular Vagus Nerve Stimulation (tAVNS) Delivered During Upper Limb Interactive Robotic Training Demonstrates Novel Antagonist Control for Reaching Movements Following Stroke

**DOI:** 10.3389/fnins.2021.767302

**Published:** 2021-11-25

**Authors:** Johanna L. Chang, Ashley N. Coggins, Maira Saul, Alexandra Paget-Blanc, Malgorzata Straka, Jason Wright, Timir Datta-Chaudhuri, Stavros Zanos, Bruce T. Volpe

**Affiliations:** ^1^Institute of Molecular Medicine, The Feinstein Institutes for Medical Research, Northwell Health, Manhasset, NY, United States; ^2^Institute for Bioelectronic Medicine, The Feinstein Institutes for Medical Research, Manhasset, NY, United States; ^3^Department of Psychiatry, University of Illinois at Chicago, Chicago, IL, United States

**Keywords:** stroke, vagus nerve stimulation (VNS), transcutaneous auricular vagus nerve stimulation (taVNS), hemiparesis, rehabilitation, robotic therapy

## Abstract

Implanted vagus nerve stimulation (VNS) delivered concurrently with upper limb rehabilitation has been shown to improve arm function after stroke. Transcutaneous auricular VNS (taVNS) offers a non-invasive alternative to implanted VNS and may provide similar therapeutic benefit. There is much discussion about the optimal approach for combining VNS and physical therapy, as such we sought to determine whether taVNS administered during robotic training, specifically delivered during the premotor planning stage for arm extension movements, would confer additional motor improvement in patients with chronic stroke. Thirty-six patients with chronic, moderate-severe upper limb hemiparesis (>6 months; mean Upper Extremity Fugl-Meyer score = 25 ± 2, range 13–48), were randomized to receive 9 sessions (1 h in length, 3x/week for 3 weeks) of active (*N* = 18) or sham (*N* = 18) taVNS (500 ms bursts, frequency 30 Hz, pulse width 0.3 ms, max intensity 5 mA, ∼250 stimulated movements per session) delivered during robotic training. taVNS was triggered by the onset of a visual cue prior to center-out arm extension movements. Clinical assessments and surface electromyography (sEMG) measures of the biceps and triceps brachii were collected during separate test sessions. Significant motor improvements were measured for both the active and sham taVNS groups, and these improvements were robust at 3 month follow-up. Compared to the sham group, the active taVNS group showed a significant reduction in spasticity of the wrist and hand at discharge (Modified Tardieu Scale; taVNS = –8.94% vs. sham = + 2.97%, *p* < 0.05). The EMG results also demonstrated significantly increased variance for the bicep peak sEMG amplitude during extension for the active taVNS group compared to the sham group at discharge (active = 26.29% MVC ± 3.89, sham = 10.63% MVC ± 3.10, mean absolute change admission to discharge, *p* < 0.01), and at 3-month follow-up, the bicep peak sEMG amplitude was significantly reduced in the active taVNS group (*P* < 0.05). Thus, robot training improved the motor capacity of both groups, and taVNS, decreased spasticity. taVNS administered during premotor planning of movement may play a role in improving coordinated activation of the agonist-antagonist upper arm muscle groups by mitigating spasticity and increasing motor control following stroke.

**Clinical Trial Registration:** www.ClinicalTrials.gov, identifier (NCT03592745).

## Introduction

Stroke is the leading cause of long-term disability in the United States (American Heart Association [AHA], 2021). Even with aggressive standard rehabilitation, more than 40% of patients experience chronic upper limb hemiparesis (Cramer et al., 1997). Recently, the combination of upper limb rehabilitation with vagus nerve stimulation (VNS) was demonstrated to improve motor outcomes in individuals with chronic post-stroke hemiparesis (Dawson et al., 2016, 2021; Kimberley et al., 2018). VNS has been shown to activate cholinergic basal forebrain, noradrenergic locus coeruleus networks important for plasticity and learning, and to enhance the release of GABA (Capone et al., 2015; Hays, 2016; Colzato and Beste, 2020), thereby potentially facilitating improvement when it is combined with motor rehabilitation.

Animal models of motor recovery following stroke have indicated specificity of recovery for only those tasks or stimuli paired with VNS. Motor behaviors paired with implanted VNS following stroke demonstrated selective increases in the size of their motor representations within the motor cortex, while motor representations for untrained tasks or tasks performed without VNS remained relatively unchanged (Porter et al., 2011; Khodaparast et al., 2014). A similar specificity of recovery for VNS-stimulated tasks has been documented in studies that focused on tinnitus reduction, in which selective pairing of VNS with tones outside of the tinnitus white noise perceptual range resulted in significant reductions in the perception of tinnitus for up to three months (Engineer et al., 2011; De Ridder et al., 2014). Taken together, these results suggest that timing, frequency, total dose delivered and characteristics of the electrical stimulation with respect to the stimulated task may all be important factors for treatment effectiveness.

In the rehabilitation of patients with chronic stroke, a notable obstacle to motor recovery is the persistence of maladaptive upper and lower limb flexor synergy patterns that impair independent control of individual joints (Zackowski et al., 2004). Some argue this aspect of the upper motor syndrome after stroke is a more significant obstacle to recovery than the traditionally defined passively elicited velocity dependent hyperactive stretch reflex (Ellis et al., 2017), commonly termed spasticity (Lance, 1980). Upper extremity flexor synergy is characterized by a fixed pattern of scapular retraction, shoulder abduction/external rotation, elbow/wrist/finger flexion, and wrist supination, resulting a “curling in” of the arm toward the body with a rigid, closed hand. It is caused by damage to the corticospinal tract and subsequent upregulation of interneuron spinal networks, and ultimately results in movement limitations, particularly for extension (McMorland et al., 2015). We have previously shown that robotic therapy provides clinically significant benefits to upper limb motor recovery after stroke (Volpe et al., 2009; Lo et al., 2010; Chang et al., 2017; Edwards et al., 2019), and can specifically reduce upper limb flexor synergy patterns through shoulder/elbow robotic training (Dipietro et al., 2007). We have also demonstrated that treatment aimed at passively elicited spasticity reduction can unmask latent motor potential (Paget-Blanc et al., 2019). In this study, we tested whether maximal and optimized current delivered to the auricular branch of the vagus nerve during pre-motor activity for robot-trained extensor movements would reduce spasticity and generate additional motor recovery of arm function after stroke.

Although many previous studies of VNS depend on invasive stimulation paired with motor training, transcutaneous auricular vagus nerve stimulation (taVNS) has emerged as a viable, efficacious, and non-invasive alternative that likely activates similar cortical networks as implanted VNS (Kraus et al., 2007; Badran et al., 2018). Here, we performed a double-blinded study using taVNS or sham stimulation paired with 3 weeks of shoulder/elbow robotic therapy. We selected a 3-week course of robotic training for study efficiency, as it has been shown to induce a reliably detectable improvement on clinical scales (Volpe et al., 2009). This study investigates whether specifically timed taVNS augments a robot trained clinical benefit and additionally produces an objective surface electromyography (sEMG) biomarker of clinical improvement in the trained muscle groups. taVNS stimulation was selectively delivered during the onset of a visual cue for extension movements to alter flexor synergy patterns and to target improved planning and execution of extension.

## Materials and Methods

### Participants

Thirty-six patients with a diagnosis of stroke and chronic (>6 months) upper limb hemiparesis were recruited by treating clinicians in the Departments of Neurology and Physical Medicine and Rehabilitation at Northwell Health (18 males, 18 females; 59.02 years of age ± 1.98, range 27.9–81.1; 2.16 years post stroke ± 0.39; Table 1). This trial was approved by the Institutional Review Board at Northwell Health, and all subjects provided written informed consent. Inclusion criteria were: (a) ≥ 18 years and ≤ 85 years of age; (b) First single focal unilateral supratentorial stroke with diagnosis verified by brain imaging (CT or MRI) that occurred at least 6 months prior; (c) Cognitive function sufficient to understand the experiments and follow instructions; (d) Upper Extremity Fugl-Meyer (UE-FM) assessment score of 12–48 points (neither hemiplegic nor fully recovered motor function in the muscles of the shoulder, elbow, and wrist). Exclusion criteria were: (a) Botox treatment within 3 months of enrollment, (b) Fixed contracture of the affected limb, (c) Complete and total flaccid paralysis of all shoulder and elbow motor performance, (d) Prior injury to the vagus nerve, (e) Severe dysphagia, (f) Introduction of any new rehabilitation interventions during study, (g) Scar tissue/broken skin at stimulation site, or irremovable metal piercings that may interfere with the stimulation or the stimulation device, (h) Highly conductive metal in any part of the body, (i) Pregnant or plan on becoming pregnant or breastfeeding during the study period, (j) Significant arrhythmias, including but not limited to, atrial fibrillation, atrial flutter, sick sinus syndrome, and A-V blocks, (k) Presence of an electrically, magnetically or mechanically activated implant, an intracerebral vascular clip, or any other electrically sensitive support system.

**TABLE 1:** Patient demographics.

Parameters (*n* = 36)	Mean (SEM)	Range
Sex, F/M, n	18/18	N/A
Age	59.02 (1.98)	27.9–81.1
Time after stroke, years	2.16 (0.39)	0.5–12.8
Type of stroke (Ischemic/Hemorrhagic)	27/9	N/A
Affected side (Dominant/Non-dominant)	17/19	N/A
Baseline Fugl-Meyer	25.27 (2.14)	13–48
Baseline MTS total upper extremity	23.5 (0.73)	12–31
Baseline MTS shoulder	7.1 (0.39)	4–12
Baseline MTS elbow	8.1 (0.27)	5–11
Baseline MTS wrist	8.6 (0.32)	3–14

A total of 144 subjects were screened for the study, and 102 subjects were excluded for the following reasons: multifocal or brainstem infarcts (42 subjects), significant cardiac arrhythmias (19 subjects), unrelated diagnosis (17 subjects), did not meet UE-FM motor inclusion criteria (8 subjects), transit issues (8 subjects), severe dysphagia (2 subjects), declined to participate (6 subjects). Thirty-six patients ultimately enrolled (Table 1). One patient dropped out for an unrelated health issue prior to completion of the intervention. One patient paused robotic intervention for greater than 3 weeks due to a family emergency, and thus this patient’s data was excluded from the analysis. Five patients were lost to follow-up (1 was unable to be reached, 2 had unrelated health issues, 2 refused to return for FU amidst COVID-19 pandemic). Thirty-four patients completed the trial through discharge and were thus included in the analysis of the immediate effects of taVNS intervention (sham taVNS = 17 subjects; active taVNS = 17 subjects). Twenty-nine patients also completed the 3-month follow-up evaluation, and were included in the measures of treatment robustness over time (sham taVNS = 15 subjects; active taVNS = 14 subjects). One patient was missing Modified Tardieu Scale (MTS) measures and one patient had corrupted follow-up EMG measurements, and thus analyses of these measures included 33 and 34 patients at discharge, respectively, and 28 patients at follow-up across both measures. All subjects were naïve to taVNS.

### Experimental Protocols

This was a double-blind, sham-controlled, repeated measures study evaluating whether 9 sessions of shoulder/elbow robotic therapy (3x/week for 3 weeks) paired with active taVNS or sham taVNS delivered during the onset of a visual cue for extension movements would significantly change objective EMG activation patterns and significantly improve clinical measures of upper extremity motor function. Patients underwent three clinical and instrumental EMG evaluations prior to intervention to verify the stability of their baseline scores. Clinical and instrumental EMG assessments were repeated immediately following 3 weeks of shoulder/elbow robotic training at discharge, and 3 months after study completion at follow-up. Upon admission, participants were classified according to baseline UE-FM with either severe (14–22 points) or moderate (23–48 points) motor impairment, and were randomized to receive active or sham taVNS, stratified by impairment level. The patient, treating clinician, and clinical evaluator were all blind to condition.

**FIGURE 1:**
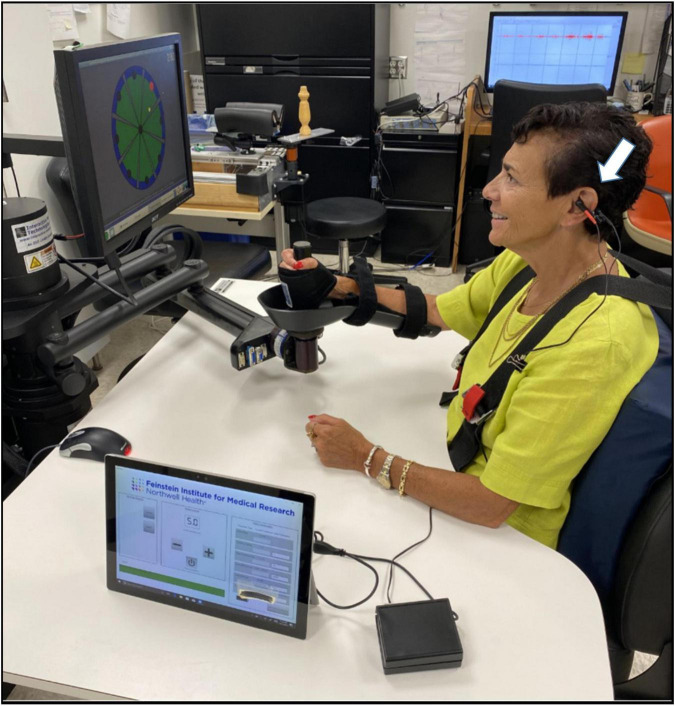
Subject receiving taVNS (arrow marks the placement of the stimulator; single 500 ms bursts, 30 Hz, pulse width = 0.3 ms, intensity just below pain threshold between 0.1 and 5.0 mA) during the blinking visual cue for the onset of extension movements on the InMotion ARM^®^ robot. During each 1 h session patients performed 1,024 active-assist center-out clock movements of the shoulder and elbow, and received stimulation during a total of 256 extensions movements (right = 9 o’clock, 10 o’clock, 12 o’clock, 2 o’clock; left = 10 o’clock, 12 o’clock, 2 o’clock, 3 o’clock).

taVNS or sham stimulation was delivered to the left cymba conchae *via* a pair of conductive silicone electrodes affixed to an ear clip, which patients wore for the duration of each active-assist robotic intervention. During each 1-h long therapy session, the patient was seated comfortably with the affected upper limb strapped into a supportive trough, and was prompted by visual cue on a computer monitor to perform a total of 1,024 center-out flexion, extension, and rotational movements of the elbow and shoulder joints (Figure 1). Robotic therapy was active-assist, such that if the patient could not move, the robot would move the patient’s arm after a 2 s delay. taVNS was delivered in single 500 ms bursts with a frequency of 30 Hz and a pulse width of 0.3 ms during the onset of the blinking visual cue for extension movements of the trained limb (right = 9 o’clock, 10 o’clock, 12 o’clock, 2 o’clock; left = 10 o’clock, 12 o’clock, 2 o’clock, 3 o’clock). A total of 256 stimulations were delivered per session. Current intensity was individually adjusted to a level just below the patient’s reported pain threshold, with amplitudes ranging from 0.1 to 5.0 mA in steps of 100 μA. A device tolerance screening questionnaire was given to all participants before and after each session. For sham stimulation, current intensity threshold was evaluated at the beginning of each session and stimulation was then ramped to zero for the duration of robot therapy. This protocol allowed sham taVNS patients to experience the sensation of treatment without delivering adequate stimulation for a therapeutic effect (Gandiga et al., 2006; Brunoni et al., 2014).

### Electrode and Stimulator Design

The transcutaneous auricular branch vagus nerve simulator device is a wireless all-in-one taVNS stimulator co-designed by engineers at the Feinstein Institutes for Medical Research and the MIDI Product Development Corporation (Smithtown, NY, United States) and fabricated by MIDI (Figure 1, arrow). It is designed to deliver low levels of current to the cymba conchae region of the ear using a pair of conductive silicone electrodes. The electrodes are affixed to a spring-load clip that is designed to fit over the left ear and are adjustable in both rotation and location, relative to the rest of the housing to accommodate subjects with different ear sizes. The device is controlled using a wireless Bluetooth link *via* an application run on a tablet that allows control over the amplitude of stimulation, onset, and timing parameters, including pulse width, frequency, burst patterns, and duration.

Photodiodes (BPW46, Vishay Intertechnology, Inc.) placed on a second robotic therapy monitor were used to detect the blinking signal for each new motor target generated by the active assist robot. Upon detection of the visual cue, a microcontroller (Arduino Leonardo; Arduino, Inc.) was used to trigger the auricular stimulation burst.

### Robotic Intervention

Robotic training was delivered with the InMotion ARM^®^ robot by Bionik Inc. The robot’s design is based on the MIT-MANUS (the planar robot), developed in the Newman Laboratory of Biomechanics and Human Rehabilitation at the Massachusetts Institute of Technology, and provides customized, goal-directed, robot assisted shoulder/elbow therapy. The hallmark of this system is an impedance controller that modulates the way the robot reacts to mechanical perturbation from a patient, and allows for a dynamic interaction, in which the patient attempts to move independently, and after a 2 s delay, the robot provides adaptive, assistance-as-needed to complete the movement (Hogan, 1985, 1988; Colgate, 1988; Volpe et al., 2009). During planar robot therapy, the patient was seated comfortably facing a computer screen with the affected hand grasping the robotic handle and the forearm gently strapped in a rigid support affixed to the robotic arm. A blinking visual cue directed the patient to reach toward points in space that corresponded to the positions of the targets on a screen, moving through over a thousand intensive, active-assist flexions, extensions, and rotational movements of the elbow and shoulder joints per session, as described in past work (Lo et al., 2010). The safety and efficacy of robotic intervention is well established and has been recognized by the American Heart Association as an effective tool for upper limb motor rehabilitation (Winstein et al., 2016).

### Instrumental Surface Electromyography Assessment

#### Setup

Electrical activity of the muscle was differentially recorded using surface EMG electrodes (Biometrics Ltd., United Kingdom) at three distinct time points: Baseline, Discharge, and Follow-up. The two electrodes were placed over the muscle belly of the biceps and triceps brachii. To ensure reproducibility of electrode placement for each patient, the length of the arm from the acromion process to the lateral epicondyle was recorded, and muscle belly of biceps and triceps brachii were palpated, with their circumferential coordinates recorded along that length. Patients were then instructed to perform maximum volitional contractions of the biceps and triceps to confirm electrode placement.

At each time point, patients performed 10 extension movements and 10 flexion movements on the robot. The first five of each acted as a warm-up; the analysis was performed on the final five movements. These movements were identical to the flexion (center-in to 6 o’clock) and extension (center-out to 12 o’clock) movements performed during robotic therapy, except that they were unassisted by the robot. For each movement, the robot would hold the patient at center in a relaxed position. The patient was then instructed to perform the extension or flexion movement without robot assistance and sEMG was recorded for each attempt. Ideally, the sEMG parameters extracted from all five movements were averaged together, however, for a small subset of noisy and inconsistent measures, fewer than five movements were accepted (this occurred in < 5% of the measures for either group).

#### Time Domain Analysis

The root mean square (RMS) was calculated and used to determine the peak amplitude of the RMS during a 2 s interval from the onset of muscle activation. To calculate muscle activation onset, a threshold was computed between 2 and 5 standard deviations, varying with each patient and muscle, from the baseline muscle activity. Among the methods used to normalize EMG recordings, we chose the isokinetic maximal voluntary contraction (MVC) which takes the peak amplitude during a dynamic movement as the reference to normalize the data (Chalard et al., 2020). The baseline activity was defined as the average of the first 500 ms of the recording while the patient was at rest, before the start of the flexion or extension task. The threshold for each individual patient was determined through visual inspection. After onset was determined, the integrated EMG (iEMG), the area under the RMS, was calculated for the 2 s time interval individually for the biceps and triceps during both flexion and extension movements. The peak amplitude of the RMS was used to derive the isokinetic maximum voluntary contraction (MVC) to normalize the data at each of the three measured timepoints (Fernández-Peña et al., 2009). The highest peak amplitude of all five flexion movements was used as the reference value for the isokinetic MVC of the biceps and, similarly, the highest peak amplitude of all five extension movements was used as the reference value for the isokinetic MVC of the triceps. Peak amplitude and iEMG are expressed as a percentage of the MVC (% MVC).

#### Frequency Domain Analysis

The EMG data was sampled at 1,000 Hz. First the data was detrended; the mean of the initial 500 ms of each frame was subtracted from the overall signal to remove any offset. To analyze the data in the frequency domain, a bandpass filter with cutoffs at 10 and 400 Hz was applied. A notch filter at 60 Hz was then applied to remove any electrical interference. The power spectral density was calculated using Welch’s method (segment length = 0.3 s and 50% overlap). The mean and median frequency were calculated from the resulting power spectral density individually for the biceps and triceps during each flexion/extension movement and are expressed in Hertz (Hz).

### Clinical Assessments

#### Upper Extremity Fugl Meyer Scale

The UE-FM is a valid and reliable assessment of performance-based impairment after stroke, measured on 0–3 ordinal scale (0 = cannot perform; 3 = performs faultlessly) with a maximum possible score of 66 points (Gladstone et al., 2002; Hsieh et al., 2009; Kim et al., 2012). The MDC (Minimum Detectable Change) is 1.56 points and the MCID (Minimal Clinically Important Difference) is 4.25 points (Page et al., 2012; Toluee Achacheluee et al., 2016).

#### Medical Research Council Motor Power Scale (MRC)

The MRC is a valid and reliable score that measures strength in isolated muscle groups of the shoulder, elbow, and wrist. It is measured on a 0–5 ordinal scale (0 = no contraction; 5 = normal strength) out of a possible 100 points (Paternostro-Sluga et al., 2008).

#### Wolf Motor Function Test

The Wolf Motor Function Test (WMFT) is a valid and reliable measure of upper limb function comprised of 15 motor-based tasks and two strength-based tasks (Wolf et al., 2001; Hsieh et al., 2009; Hodics et al., 2012). It is scored on both a Functional Ability Scale (WOLF-FAS) to measure the quality of the movement (0–5 ordinal scale out of 75 possible points) and a time test (WMFT time) to measure the speed of the movement (up to 120 s per task out of a maximum 1,800 s). Improvement is reflected by an increase in WOLF-FAS score and a decreased in WMFT time.

#### Modified Tardieu Scale

The MTS is a valid and reliable measure of spasticity to passive movement at slow (V1) and fast (V2) speeds (Paulis et al., 2011; Singh et al., 2011). Each joint is measured on a 0–5 ordinal scale (0 = no resistance, 5 = joint immobile), with higher scores indicating increased spasticity. Given that robotic intervention targeted more than one joint of the upper limb, the MTS was evaluated both as a summed score across 11 joints of the upper extremity, MTS total, and at the individual joint complexes for the shoulder, MTS shoulder (summed across 3 joints: horizontal adductors, vertical adductors, internal rotators), the elbow, MTS elbow (summed across 4 joints: elbow flexors, elbow extensors, pronators, supinators), and the wrist, MTS wrist (summed across 4 joints: wrist flexors, wrist extensors, fingers, palmer interrossei/flexor digitorum superficilias). For studies involving whole-limb intervention, summed scores are advantageous as they may more sensitively detect changes across trained muscles groups (Pundik et al., 2014; Paget-Blanc et al., 2019). We selected to use the MTS instead of the Modified Ashworth Scale (MAS) as the MTS has been shown to be more sensitive to changes in spasticity (Mehrholz et al., 2005; Haugh et al., 2006). For MAS, the minimal clinically important difference (MCID) for a single joint is defined as a 1-point reduction (Brashear et al., 2002). As no MCID is established for summed measurements on either the MAS or MTS, we defined a response to treatment as at least a 2-point reduction for any single joint complex (shoulder/elbow/wrist), and at least a 3-point reduction the MTS total score summed across the whole upper limb.

### Statistical Analysis

Statistical analysis was performed using Sigmaplot version 14.5. Acute effects of the intervention at discharge (*N* = 34) and robustness of the treatment effect at follow-up (*N* = 28) were analyzed separately. For normally distributed data, a Welch’s *t*-test was used to compare between-groups (active vs. sham) changes from baseline to discharge (D-A) and baseline to follow-up (F-A), respectively, and One-way RM-ANOVA was used to analyze within-group changes over time (admission, discharge, follow-up). For data that violated the assumptions of parametric statistics, non-parametric comparisons were made using Mann-Whitney *U* Tests to examine between-groups changes from baseline at discharge (D-A) and follow-up (F-A), and Friedman RM-ANOVA was used to analyze within-group changes over time (admission, discharge, follow-up). *Post hoc* Tukey tests for multiple comparisons were applied as warranted. Results are presented as mean ± standard error of the mean (SEM), unless otherwise specified.

## Results

### Clinical Outcomes for Motor Function

**FIGURE 2:**
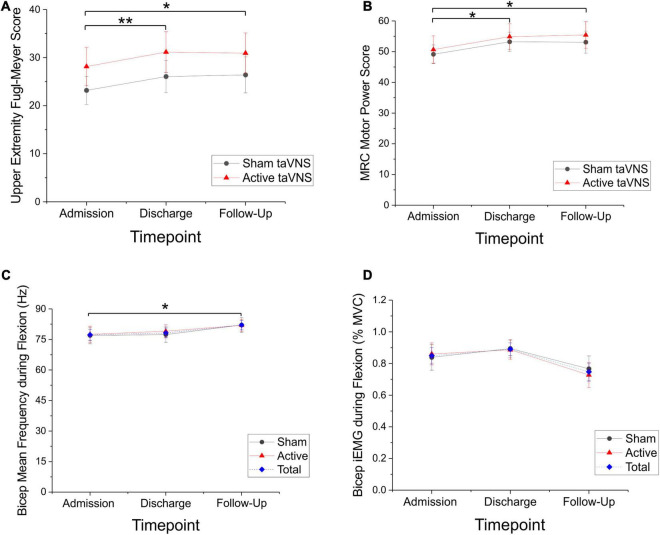
Significant motor improvements and significant changes in sEMG measures of bicep mean frequency were seen in both the active and sham taVNS groups, suggestive of a training benefit from the robot. **(A)** Upper Extremity Fugl Meyer scores (mean ± SEM) improved for both the sham (*N* = 15) and active (*N* = 14) taVNS groups, with a mean improvement of 3 points (Friedman RM-ANOVA, sham *P* < 0.001, Chi-square = 20.920; active *P* < 0.001, Chi-square = 16.453). Improvements were significant at discharge and robust through follow-up (Tukey test, sham and active: adm-dc **P < 0.001, adm-fu *P < 0.01). **(B)** MRC motor power scores (mean ± SEM) were also improved for both the sham (*N* = 15) and active (*N* = 14) groups (Friedman RM-ANOVA, sham *P* < 0.01, Chi-square = 13.0; active *P* < 0.001, Chi-square = 15.434). These improvements were significant at discharge and robust through follow-up (Tukey test, active and sham: adm-dc *P ≤ 0.01; sham: adm-fu *P < 0.05, active adm-fu *P < 0.001). **(C)** Directional trends for sEMG are significant for both summed sham and active groups (mean ± SEM; *N* = 28; blue dashed line). Mean frequency of the biceps during flexion significantly increased across the combined group (One-way RM-ANOVA, *P* < 0.05, *F* = 3.274), between admission and follow-up (Tukey test, *P < 0.05). **(D)** Biceps iEMG (area under the RMS curve) during flexion approached a significant reduction (in% mean voluntary contraction) across the combined group (One-way RM-ANOVA, *P* = 0.050).

There were significant motor improvements after robotic training for both sham and active taVNS groups, and these improvements were robust at follow-up. Specifically, UE-FM scores improved for each group (Friedman RM-ANOVA, sham *P* < 0.001, Chi-square = 20.920; active *P* < 0.001, Chi-square = 16.453). *Post hoc* pairwise comparisons were significant from admission to discharge and admission to follow-up for both sham and active groups (Tukey test, sham and active: adm-dc *P* < 0.001, adm-fu *P* < 0.01; Figure 2A). Average improvement on the UE-FM was approximately 3 points for the active and sham groups at discharge (sham = 2.86 ± 0.50; active = 3.10 ± 0.57) and follow-up (sham = 3.22 ± 1.0; active = 2.79 ± 0.84), which is a reliable improvement above the minimum detectable change (MDC) of 1.56 points, but less that the minimal clinically important difference (MCID) of 4.25 points.

Similarly, MRC motor power scores improved for both groups (Friedman RM-ANOVA, sham *P* < 0.01, Chi-square = 13.0; active *P* < 0.001, Chi-square = 15.434). *Post hoc* pairwise comparisons showed significant improvements from admission to discharge and admission to follow-up for both the sham and active groups (Tukey test, active and sham: adm-dc *P* ≤ 0.01; sham: adm-fu *P* < 0.05, active adm-fu *P* < 0.001; Figure 2B). Average improvement on the MRC was 4 points at discharge (sham = 4.00 ± 0.87, active = 4.07 ± 0.63) and approximately 4–5 points at follow-up, (sham = 3.69 ± 1.33, active = 4.56 ± 1.15).

Finally, Wolf FAS score improved within each group (Friedman RM-ANOVA, sham: *P* < 0.05, Chi-Square = 8.821; active: *P* < 0.01, Chi-square = 9.542), and *post hoc* pairwise comparisons revealed this change only occurred from admission to follow-up (Tukey test, sham and active: adm-fu, *P* < 0.05; sham = 2.53 ± 0.9, active = 3.00 ± 1.04). Wolf time score decreased within each group, as patients improved and performed functional tasks faster (Friedman RM-ANOVA, sham: *P* < 0.05, Chi-square = 6.933; active: *P* < 0.01, Chi-square = 11.236). *Post hoc* pairwise comparisons were significant for the active group only from admission to discharge, and the sham and active groups from admission to follow-up (Tukey test, active: adm-dc *P* < 0.05; sham: adm-fu *P* < 0.05, active: adm-fu *P* < 0.01).

Change scores were not different between active and sham taVNS groups at discharge or follow-up for the UE-FM Scale, MRC, Wolf FAS score, or Wolf time score (Mann-Whitney *U*-test, *P* ≥ 0.230 across groups).

Tolerance of the stimulation was assessed in questionnaires and the stimulation was well tolerated with no differences reported across the sham and treated groups. The current was set to be less than the pain threshold, and this maneuver rendered the stimulation non-toxic, well-tolerated and not overtly distracting.

### Clinical Spasticity Outcomes: Modified Tardieu Scale

**FIGURE 3:**
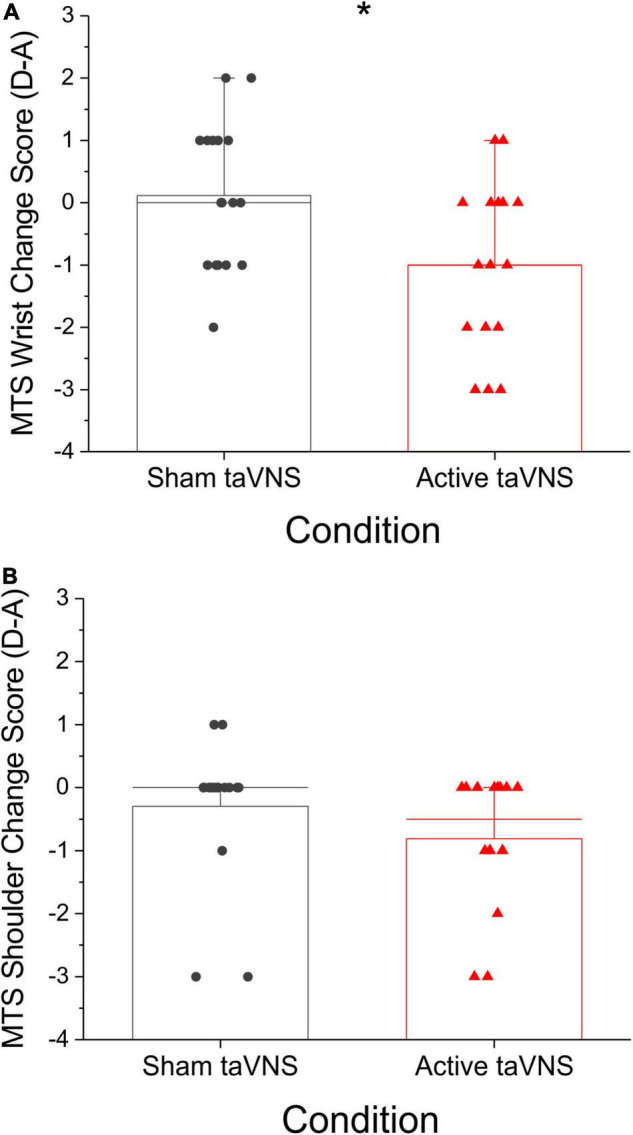
taVNS delivered during robotic therapy extension movements significantly reduced spasticity for the active taVNS group (*N* = 16) compared to sham (*N* = 17) at discharge (mean raw change score ± SEM), but not follow-up (data not shown). Lower (more negative) scores indicate a greater reduction in spasticity. **(A)** MTS Wrist/Hand change score at discharge was significantly reduced for the active group (*P < 0.05, Mann-Whitney *U*-test, *U* = 77.0; sham = + 0.17 ± 0.26, active = –0.79 ± 0.31). **(B)** MTS shoulder change score at discharge approached a significant difference for the active taVNS group (*P* = 0.051, *U* = 88.50, Mann-Whitney *U*-test, sham = –0.25 ± 0.24, active = –0.72 ± 0.25).

Patients receiving active taVNS during shoulder/elbow robot training had a significant decrease in the MTS wrist score at discharge compared to patients receiving the sham treatment *(P* < 0.05, Mann-Whitney *U*-test, *U* = 77.0; sham = + 0.17 ± 0.26, active = –0.79 ± 0.31; Figure 3A). This difference was not apparent at follow-up (*P* = 0.207). On closer analysis, the Modified Tardieu measure of spasticity for the wrist demonstrated a significant difference for the active taVNS treated group (Friedman RM-ANOVA sham: *P* = 0.420; active: *P* < 0.05, Chi-square = 6.588). MTS total score and MTS shoulder score also decreased in the active compared to the sham condition, and these changes approached significance at discharge (MTS total: *P* = 0.0616, *t* = 1.945, Welch’s *t*-test, sham = –0.49 ± 0.38, active = –1.60 ± 0.50; MTS shoulder: *P* = 0.051, U = 88.5, Mann-Whitney U-test, sham = –0.25 ± 0.24, active = –0.72 ± 0.25; Figure 3B). MTS scores measured at the elbow showed no significant change. Using a 2-point reduction in the MTS for a single joint complex as significant, responder rates at discharge for the wrist were 5.9% (1/17) for sham and 37.5% (6/16) for active taVNS, and for the shoulder were 11.8% (2/17) for sham and 18.8% (3/16) for active taVNS. Using a 3-point reduction in the MTS total as significant, responder rates as discharge were 11.8% (2/17) for sham and 33.3% (5/16) for active taVNS.

### Objective Surface Electromyography Outcomes: Muscle Activation

#### Bicep Surface Electromyography Changes During Extension Movements

**FIGURE 4:**
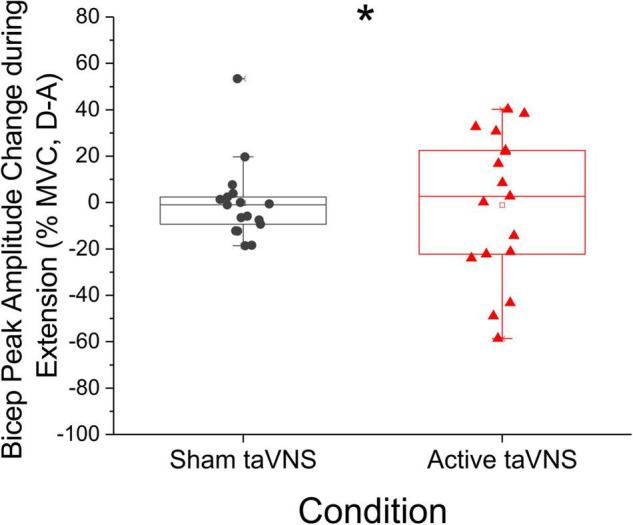
Peak amplitude change score in antagonist (biceps) muscles during extension was notably more dispersed in active taVNS group (*N* = 17) compared to the sham group (*N* = 17) at discharge (% mean voluntary contraction change score ± SEM). The change in bicep peak RMS amplitude during extension movements was not significantly different between active and sham groups at discharge or follow-up (Mann-Whitney *U*-test, discharge: *P* = 0.796, *U* = 137.0; follow-up: *P* = 0.183, *U* = 69.0), but change score variance was significantly different between groups at discharge (Siegel-Tukey test, *P < 0.01).

**FIGURE 5:**
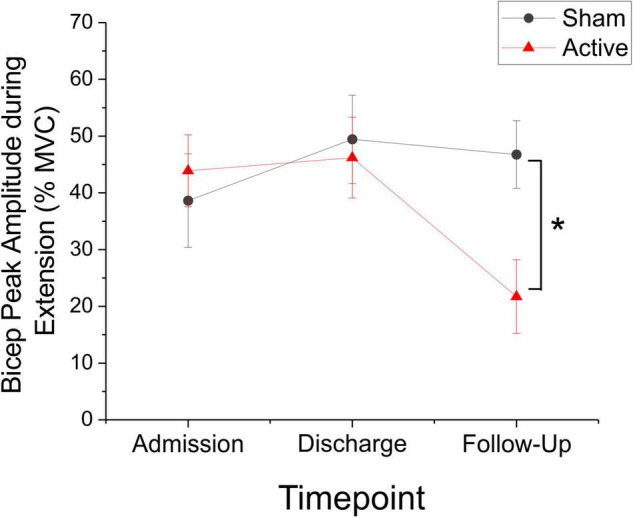
Bicep Peak amplitude (as represented by% mean voluntary contraction ± SEM) decreased significantly during extension movements in the active taVNS group (*N* = 14), but not the sham group (*N* = 14) at follow-up (Friedman RM-ANOVA, sham: *P* = 0.931; active: *P* < 0.05, Chi-square = 7.0). *Post hoc* pairwise comparisons showed a significant reduction in bicep peak RMS amplitude between discharge and follow-up for the active taVNS group (Tukey test, *P < 0.05). There were no significant between-groups differences.

Although the change in bicep peak RMS sEMG amplitude during extension movements was not significantly different between active and sham groups at discharge or follow-up (Mann-Whitney *U*-test, discharge: *P* = 0.796, *U* = 137.0; follow-up: *P* = 0.183, *U* = 69.0), *post hoc* comparisons of change score variance revealed significant between-group differences in the dispersion of the data at discharge (Siegel-Tukey test, *P* < 0.01; mean absolute change admission to discharge, sham = 10.63 ± 3.10, active = 26.29 ± 3.89; Figure 4). Within groups, Friedman RM-ANOVA revealed that bicep peak RMS amplitude during extension was significantly reduced for the active condition only (Friedman RM-ANOVA, sham: *P* = 0.931; active: *P* < 0.05, Chi-square = 7.0; Figure 5). *Post hoc* pairwise comparisons showed a significant reduction in bicep peak RMS amplitude between discharge and follow-up for the active taVNS group (Tukey test, *P* < 0.05).

There were no significant changes in the biceps iEMG or mean/median frequency during extension. There were also no significant changes across all measures for the triceps or the ratio of the biceps to triceps during extension.

#### Bicep Surface Electromyography Changes During Flexion Movements

When all patients (sham and active taVNS) were combined into a single group and the effect of time was assessed, there was a significant increase in bicep mean frequency during flexion (One-way RM-ANOVA, *P* < 0.05, *F* = 3.274; Figure 2C) and a reduction in bicep iEMG during flexion, which approached significance (One-way RM-ANOVA, *P* = 0.050; Figure 2D). *Post hoc* pairwise comparisons revealed a significant increase in bicep mean frequency between admission and follow-up for the combined (active and sham) group (Tukey test, *P* < 0.05). Both sham and active taVNS groups trended in the same directions for these measures, but neither reached significance independently.

There were no significant changes in the biceps median frequency, the ratio of biceps to triceps, or in the triceps across all sEMG measures during flexion.

### Stimulation Safety and Device Tolerance

Stimulation was well-tolerated and there were no serious adverse events. The average tolerated intensity of taVNS current was 4.5 mA ± 0.06 (mean ± SEM).

## Discussion

The present study demonstrates that taVNS delivered prior to extension movements in a shoulder/elbow robotic training task significantly reduced spasticity in the affected arm, and significantly changed bicep peak sEMG amplitudes during extension. Motor improvements, on all clinical scales, were significant for both the active and sham taVNS groups and robust through follow-up and are indicative of a benefit from robot training. Using an MCID of a 2-point or greater reduction in spasticity, improvements in the wrist and hand were clinically significant for more than a third (6/16 = 37.5%) of the active taVNS group compared with 5.9% (1/17) of the sham group after the training period, though this improvement, unlike the motor improvements, was not maintained in follow-up. The decreased spasticity measure at discharge was an unexpected result, given that robot training was focused on the shoulder and elbow. It may be that the requirement for robotic training that places the hand around a joystick to perform shoulder/elbow movements is similar to a splinted stretch treatment and may have contributed to a relaxing of muscles in the distal forearm. Nonetheless, spasticity improvements were significant only for the active group, suggestive that taVNS targeted to extensor movements augments reduction of spasticity.

Objective sEMG measures of bicep peak amplitude during extension were significantly different for the active treatment group, however, neither this peak amplitude nor the increased variance of the peak amplitude at discharge and its resolution at follow-up, led to differential motor improvement. sEMG measures of the triceps and any of the reciprocal relationships to biceps sEMG were also unrevealing. Others have reported abnormal and unpredictable antagonist-agonist relationships in patients recovering from stroke (Burke, 1988) that, at times, correlated with stroke severity (Levin et al., 2018). Nevertheless, the EMG findings in the antagonist biceps of the treated group, begs the question of whether a longer treatment stimulation period or a higher or more frequent dose of stimulation would have led to a separation of motor performance between the groups.

Motor improvements on the UE-FM, the MRC motor power scale, and the Wolf Motor Function Test were significant for both the sham and active taVNS groups and robust through follow-up. The average UE-FM improvement was 3 points across both groups at discharge and follow-up, which is above the MDC, indicating a reliably measured improvement, but is below the MCID threshold of 4.25 points that have been taken to indicate a functionally significant change. Similarly for the objective EMG measures, when the active and sham taVNS groups were combined, there was a significant increase in the mean frequency of the biceps during flexion and a reduction of the iEMG that approached significance. Consequently, these results present a potential dichotomy for future taVNS studies between sEMG measures of general motor improvements in comparison to early biomarkers of distinct motor change attributed to taVNS.

Unlike the results in our study, other trials of VNS-paired motor training following stroke have reported significant improvements in UE-FM measures for those treated with VNS (Capone et al., 2017; Dawson et al., 2021). The difference appears to depend on increased cumulative dose of stimulation and training, and the implanted stimulator may have additional advantages (Dawson et al., 2021). Specifically, compared to 9 training sessions and ∼250 stimulated movements per session in the present study, Dawson et al. report that patients received 18 sessions (3x/wk for 6 weeks) of in-clinic therapy paired with > 300 stimulated movements (0.8 mA, 30 Hz) per session. Additionally, that trial continued with a 30-min/day home exercise program coupled with continuous VNS delivered every 10 s for 30 min until the 90-day follow-up. In another study that employed taVNS also using higher doses of stimulation, Capone et al. (2017) report that patients received 10 consecutive daily taVNS sessions in a single block that delivered pulse trains lasting 30 s (0.8 mA, 30 Hz), every 5 min for 1 h, prior to wrist or shoulder/elbow training. Thus, the combined results suggest that patients in our study were undertreated.

The novelty of the present study was the selectivity of current delivery in a closed-loop during visual cues for active-assist extension movements. Although the higher doses of stimulation and the extended treatment in other studies trumped treatment timing, it remains remarkable, given the low dose of stimulation and short duration of intervention in our study, that patients in the active taVNS group showed distinct improvements in upper limb spasticity of the wrist and hand at discharge and greater changes in bicep peak sEMG amplitude for trained extension movements. This suggests that selection of impairment-focused motor targets (e.g., extension movements) with taVNS may improve efficiency of training. Future studies of taVNS targeted to impairment-focused training should be longer duration, with a higher dose of stimulation to determine if changes in antagonist control may induce functional improvements.

## Conclusion

Our results showed that 3 weeks of upper limb robotic training combined with taVNS delivered selectively during extension movements demonstrated significant reductions in spasticity at the wrist and hand and significant changes in bicep sEMG peak amplitude during extension movements. Similar improvements in clinical scales were seen in both active and sham groups. Changes in bicep peak sEMG amplitude may be a sensitive early biomarker of taVNS-induced improvements.

## Data Availability Statement

The raw data supporting the conclusions of this article will be made available by the authors, without undue reservation.

## Ethics Statement

The studies involving human participants were reviewed and approved by Institutional Review Board at Northwell Health. The patients/participants provided their written informed consent to participate in this study. Written informed consent was obtained from the individual(s) for the publication of any potentially identifiable images or data included in this article.

## Author Contributions

JC, MSa, AP-B, AC, and BV designed the study. MSt, JW, and TD-C contributed to the design of the taVNS stimulation and current delivery software. JC, MSa, AP-B, and AC performed research. JC, AC, and BV analyzed, interpreted data, and wrote the article. MSa, AP, MSt, JW, TD-C, and SZ provided additional comments and contributed to finalizing the article. All authors read and approved the final manuscript.

## Conflict of Interest

The authors declare that the research was conducted in the absence of any commercial or financial relationships that could be construed as a potential conflict of interest.

## Publisher’s Note

All claims expressed in this article are solely those of the authors and do not necessarily represent those of their affiliated organizations, or those of the publisher, the editors and the reviewers. Any product that may be evaluated in this article, or claim that may be made by its manufacturer, is not guaranteed or endorsed by the publisher.
